# Early discharge as a mediator of greater ICU‐level care requirements in patients not enrolled on the AAML0531 clinical trial: a Children's Oncology Group report

**DOI:** 10.1002/cam4.839

**Published:** 2016-07-29

**Authors:** Kelly D. Getz, Tamara P. Miller, Alix E. Seif, Yimei Li, Yuan‐Shung Huang, Todd Alonzo, Robert Gerbing, Lillian Sung, Matthew Hall, Rochelle Bagatell, Alan Gamis, Brian T. Fisher, Richard Aplenc

**Affiliations:** ^1^ The Children's Hospital of Philadelphia3535 Market StreetPhiladelphiaPennsylvania19104; ^2^ The Children's Hospital of Philadelphia3501 Civic Center BoulevardPhiladelphiaPennsylvania19104; ^3^ University of Southern CaliforniaChildren's Oncology Group222 E. Huntington DriveMonroviaCalifornia91016; ^4^ Children's Oncology Group222 E. Huntington DriveMonroviaCalifornia91016; ^5^ The Hospital for Sick Children555 University AvenueTorontoOntarioCanada; ^6^ Children's Hospital Association6803 W. 64th StreetOverland ParkKansas66202; ^7^ Children's Mercy Hospitals and Clinics2401 Gillham RoadKansas CityMissouri64108

**Keywords:** Acute myeloid leukemia, disparities, mediation, patient discharge, pediatrics, trial enrollment

## Abstract

Previous data suggest that patients enrolled on clinical trials for treatment of cancer have better overall survival than patients who do not enroll; however, short‐term outcomes relative to trial enrollment and corresponding mediators have not been assessed. A cohort of pediatric patients with newly‐diagnosed acute myeloid leukemia was assembled from the Pediatric Health Information System. We evaluated whether patients not enrolled onto Children's Oncology Group trial AAML0531 had greater intensive care unit (ICU)‐level requirements than enrolled patients and whether early discharge after chemotherapy administration mediated this association. Patients not enrolled on AAML0531 were more likely to be discharged early (aOR = 1.40, 95% confidence interval [CI]: 1.02, 1.90) and to require ICU‐level care (aOR = 2.00, 95% CI: 1.06, 3.78) than enrolled patients, but early discharge explained only a small proportion (12.3%) of the absolute difference in ICU‐level care risk. The direct effect of nonenrollment on the need for ICU‐level care was significant (aOR = 1.89, 95% CI: 1.00, 3.94), whereas the indirect effect mediated through early discharge was not (aOR = 1.07, 95% CI: 0.95, 1.19). Factors other than postchemotherapy discharge strategy drive the difference in ICU utilization by trial enrollment status.

## Introduction

There is conflicting evidence for a direct beneficial effect of clinical trial participation on treatment outcomes among patients with cancer 1, 2. Trial enrollment may improve short‐ and long‐term outcomes for several reasons, most obviously from the benefit of the experimental treatment. However, other differences in the care of participants relative to nonparticipants, particularly supportive care strategies, may mediate the benefit associated with enrollment.

We recently evaluated associations between early discharge to outpatient management during postchemotherapy neutropenia (relative to inpatient management) and both utilization of intensive care unit (ICU)‐level care 3 and incidence of toxicities 4 using two different newly‐diagnosed pediatric acute myeloid leukemia (AML) patient populations. The results were complementary and suggested that increases in ICU requirements observed following early discharge were largely due to infectious complications. However, the observed rates of early discharge differed between the two studies. The primary difference between the studies: one included only patients enrolled on Children's Oncology Group (COG) trial AAML0531 (rate of early discharge ~8.5%) and the other included patients receiving standard chemotherapy at free‐standing children's hospitals across the US, irrespective of trial enrollment (rate of early discharge ~19%).

Given these observations and literature suggesting differential outcomes among cancer patients by trial enrollment, we evaluated whether patients not enrolled onto AAML0531 had greater ICU utilization than those enrolled. We then assessed whether this association was mediated by differences in postchemotherapy discharge strategy.

## Materials

### Study population

The study population was derived from a previously assembled cohort of children who received standard frontline chemotherapy for AML in the Pediatric Health Information System administrative billing database (PHIS) 3, 5. Daily pharmacy data for each patient were manually reviewed and chemotherapy administration patterns were matched to conventional pediatric AML treatment regimens. We restricted the entire study population to patients who were reliably identified as having received the same standard AML chemotherapy backbone as used in AAML0531 (Fig. S1). Specifically, the analyses were limited to patients who received the following regimens: ADE (cytarabine, daunorubicin, etoposide, ±gemtuzumab) at induction courses, AE (cytarabine, etoposide) at Intensification I, and MA (mitoxantrone, cytarabine, ±gemtuzumab) at Intensification II. If a patient received chemotherapy that was inconsistent with the regimens defined above, the patient was excluded from the analyses for that course and any subsequent courses. The cohort was limited to patients treated at AAML0531‐participating hospitals, and who received their first induction chemotherapy course during the period when AAML0531 was open and enrolling patients, 2006–2010 6. Hospitalizations included in the analysis were those in which patients were determined to be eligible for early discharge upon completion of a chemotherapy course 3. A patient was considered early discharge‐eligible if there was no record of ICU‐level care or blood culture from the first day of chemotherapy through the last day of chemotherapy plus 3 days. ICU‐level care was defined by the occurrence of specific International Classification of Diseases‐Ninth Revision‐Clinical Modification (ICD‐9‐CM) procedure codes or clinical resources considered a priori as a marker of ICU care, rather than by physical location 7. PHIS data were merged with data from the AAML0531 trial as previously described 8.

### Exposure

The exposure of interest was enrollment status on AAML0531. Patients identified in both the COG and PHIS datasets were considered “enrolled” while patients with only PHIS data were considered “not enrolled”. ICD‐9‐CM diagnosis codes were reviewed to confirm that “not enrolled” patients would not have violated AAML0531 exclusion criteria.

### Outcome

Course‐specific follow‐up started 4 days after the last day of chemotherapy and continued until the start of the subsequent course, death, or 50 days after commencement of the last chemotherapy course, whichever occurred first. The primary outcome of interest was receipt of ICU‐level care (any, none) over the duration of follow‐up.

Utilization rates (days of use per 1000 inpatient days) for each of the following resources were also presented to explore the nature of the specific ICU requirements: antibiotic, antifungal, antiviral, and vasopressor medications, parenteral nutrition, blood product replacement, and supplemental oxygen.

### Proposed mediator

Early discharge‐eligible patients were categorized based on timing of discharge relative to the last day of chemotherapy. Patients discharged within 3 days after chemotherapy completion were categorized as “early discharge” and all others were categorized as “standard discharge”.

### Statistical methods

We proposed a mechanistic model of the association between trial enrollment status and ICU‐level care requirements suggesting postchemotherapy neutropenia management strategy (early vs. standard discharge) as a possible mediator. The following are required for early discharge to be a potential mediator: significant associations between (1) trial nonenrollment and early discharge and (2) early discharge and ICU‐level care, and (3) a non‐null relationship between trial nonenrollment and ICU‐level care 9, 10. In order to estimate the impact of early discharge as a mediator of the association between nonenrollment and ICU‐level care, we first assessed the requisite component associations. Odds ratios (OR) and 95% confidence intervals (CI) for the effects of trial enrollment on early discharge and ICU‐level care and the effect of early discharge on ICU‐level care were estimated using logistic regression. Estimates from the model for ICU‐level care conditional on trial enrollment and early discharge, and from the model for early discharge conditional on trial enrollment were used to compute the indirect (as mediated by early discharge) and direct (through other undefined pathways) effects of trial enrollment on the risk for ICU‐level care 9, 10. Crude and adjusted analyses were completed including an exposure‐mediator interaction. Poisson regression models with log‐transformed inpatient days as offset were used to estimate adjusted rate ratios (aIRR) and 95% CI for the effect of enrollment on resource utilization; a Pearson scaler was used to correct possible overdispersion. General estimating equation methods with an exchangeable correlation matrix were utilized to account for correlation between observations within an individual. All multivariable adjusted models included control for the following potential confounders: course, age, sex, race, insurance status, and gemtuzumab exposure. All analyses were performed using SAS (version 9.2, SAS Institute, Inc., Cary, NC).

All patients enrolled on AAML0531 provided informed consent for use of trial data for research. All patient data remained deidentified throughout data merging and analysis.

## Results

The current analyses included 941 chemotherapy courses contributed by 390 pediatric AML patients. There were no significant differences in distributions of race, gender, age at diagnosis, or insurance at diagnosis between patients who were enrolled on the clinical trial and those who were not (Table 1).

**Table 1:** Covariate distribution by AAML0531 trial enrollment

Characteristics	Enrolled (*n* = 243) ( %)	Not enrolled (*n* = 147)	*χ* ^*2*^ *P*‐value
Race			0.207
White	146 (59.9)	97 (66.3)	
Non‐White	97 (40.1)	50 (33.7)	
Gender			0.223
Female	139 (57.0)	74 (50.6)	
Male	104 (43.0)	73 (49.4)	
Age, years			0.126
0–1	27 (11.3)	18 (12.4)	
1–5	63 (26.1)	38 (25.5)	
5–10	41 (16.9)	21 (14.4)	
10–15	81 (33.1)	37 (25.1)	
15^+^	31 (12.7)	33 (22.6)	
Insurance type			0.500
Private	92 (38.0)	54 (36.6)	
Public	96 (39.4)	66 (44.9)	
Other	55 (22.5)	27 (18.5)	

First, we evaluated whether trial enrollment was independently associated with the proposed mediator, early discharge. Patients not enrolled on AAML0531 were significantly more likely to be discharged early than the patients who were enrolled (aOR = 1.40, 95% CI: 1.02, 1.90) (Table 2). Next, we evaluated whether the proposed mediator, early discharge, was also independently associated with the outcome of interest. Patients discharged early were more likely to require ICU‐level care than standard discharge patients (aOR: 2.00, 95% CI: 1.06, 3.78). Finally, we considered whether trial enrollment was independently associated with the outcome of interest, ICU‐level care. Patients who were not enrolled were significantly more likely to require ICU‐level care than those enrolled on AAML0531 (aOR = 2.06, 95% CI: 1.06, 4.01).

**Table 2:** Comparison of early discharge and ICU‐level care by AAML0531 trial enrollment status

	AAML0531 Trial enrollment		Unadjusted odds ratio	Adjusted^a^ odds ratio
	Not enrolled	Enrolled	(95% CI)	(95% CI)
ICU‐level care, *n* (%)	19 (7.7)	6 (4.2)	1.90 [1.02, 3.54]	2.00 [1.06, 3.78]
Early discharge, *n* (%)	96 (39.6)	42 (29.1)	1.39 [1.04, 2.12]	1.40 [1.02, 1.90]

Reference = AAML0531 enrolled.

a Adjusted for course, age at diagnosis, race, gender, insurance at course start, and gemtuzumab exposure.

Because all three criteria were met to consider early discharge as a potential mediator, we decomposed the total effect of trial enrollment (not enrolled vs. enrolled) on the risk for ICU‐level care into its indirect effect through early discharge (aOR: 1.07, 95% CI: 0.95, 1.19) and its direct effect (aOR: 1.89, 95% CI: 1.00, 3.94) through other undefined pathways. Altogether, 12.3% of the absolute effect of trial enrollment on ICU‐level care was mediated through early discharge.

With respect to the specific pattern of resources utilized, patients not enrolled on AAML0531 incurred higher rates of vasopressor (aIRR: 2.30, 95% CI: 1.03, 5.15), fresh frozen plasma (aIRR: 3.15, 95% CI: 1.23, 8.05), and supplemental oxygen (aIRR: 2.79, 95% CI: 1.42, 5.47) than patients enrolled on the trial (Table 3).

**Table 3:** Comparisons of resource utilization rates (per 1000 inpatient days) by AAML0531 trial enrollment status

	AAML0531 enrollment		aIRR^a^ (95% CI)
	No	Yes	
Antibiotics	1163.2	1110.6	1.05 [0.98, 1.17]
Antifungals	876.8	845.8	1.04 [0.98, 1.09]
Antivirals	123.5	116.2	1.10 [0.77, 1.58]
Vasopressors	23.7	9.9	2.16 [1.15, 4.09]*
Blood products, total	316.0	299.9	1.03 [0.92, 1.16]
Platelets	185.7	173.4	1.05 [0.93, 1.19]
Packed RBC	123.3	124.0	0.98 [0.85, 1.13]
Fresh frozen plasma	5.0	1.5	3.15 [1.23, 8.05]*
Parenteral nutrition	129.2	126.8	1.07 [0.74, 1.54]
Oxygen therapy	26.6	10.3	2.79 [1.42, 5.47]*

aIRR, adjusted utilization rate ratio; reference = AAML0531 enrolled.

a Adjusted for course, age at diagnosis, race, gender, insurance at course start, and gemtuzumab exposure.

indicates p‐value <0.05.

## Discussion

This study found a higher risk of both ICU‐level care and early discharge for patients not enrolled on AAML0531. Because we previously demonstrated that patients discharged early to outpatient management also sustained greater rates of ICU‐level care a difference which was driven primarily by increased rates of bacterial infections 3, we postulated that early discharge could be a mediator of the association between enrollment status and the increased ICU‐level care requirements. This hypothesis was further supported by the fact that COG supportive care guidelines discourage early discharge by recommending hospitalization following AML chemotherapy until neutropenia recovery. Our analyses demonstrate that all three criteria were met to establish early discharge as a potential mediator of the association between trial enrollment status and the subsequent requirement for ICU‐level of care. However, when the association was decomposed into its natural indirect effect through discharge strategy and its natural direct effect through other pathways, only a small proportion (12%) of the absolute increase in the risk for ICU‐level care relative to non‐enrollment was through early discharge. In this study, patients not enrolled on AAML0531 incurred higher rates of vasopressor, fresh frozen plasma, and supplemental oxygen than enrolled patients, but had similar rates of antibiotic utilization. Thus, it is not surprising that early discharge was found to be a weak mediator of the observed association between trial enrollment and the risk for ICU‐level care.

Together, these results suggest that factors other than postchemotherapy discharge strategy explain the increase in ICU‐level care requirements among those not enrolled on AAML0531. In adult studies of various malignancies, trial participants have been shown to be a prognostically more favorable subset of patients 1, thus a viable alternative explanation for the observed disparity in ICU‐level care requirements may be differences in clinical status at presentation between patients who enroll and who do not enroll on clinical trials. A more acute clinical status at presentation, whether it be due to underlying disease biology or delays in accessing care, would limit the time and potentially the comfort of the treating clinician and/or the patient and parent to consider enrollment on an experimental trial and also be associated with an increased toxicity risk leading to a more favorable subset of patients being enrolled.

We employed mediation methods which allowed for control for confounding by a variety of covariates and incorporation of an interaction between the exposure and mediator 9, 10. To limit the heterogeneity between compared groups, we also restricted the study population of both enrolled and not enrolled patients to discharge‐eligible patients who received the same standard chemotherapy backbone in each treatment course, and reviewed ICD‐9 diagnosis codes from the first admission onwards to confirm that all patients included in the nonenrollment population did not meet an AAML0531 exclusion criterion in an attempt. Still, we cannot rule out the possibility of residual confounding by unmeasured factors. Unfortunately, data available in PHIS do not include laboratory test results thus precluding the evaluation of biologic factors that could serve as markers for potential confounders such as baseline acuity, or mediators to the associations of interest, such as absolute neutrophil count recovery following chemotherapy. Likewise, comorbidities and socioeconomic factors are not able to be fully evaluated using the inpatient data available in PHIS. Many hospitals in the United States have Day Hospital models, the existence of which could presumably lead to greater use of early discharge and to lower rates of ICU‐level inpatient care among those patients discharged prior to count recovery, but this practice is not captured in PHIS. Such confounding of the mediator‐outcome association may have resulted in an underestimation of the indirect effect of nonenrollment on ICU‐level care. Lastly, in the absence of data on the exact enrollment periods for each participating site, we utilized the broader window during which any enrollment occurred to define our AAML0531‐eligible study population which may have resulted in a bias of the observed associations toward no effect.

These analyses suggest that pediatric AML patients not enrolled on clinical trials have poorer short‐term outcomes, specifically greater ICU requirements, than those enrolled. Additionally, the proposed mediator, early discharge, explained only a small proportion of the difference in outcomes by trial enrollment status suggesting that changes in this practice are unlikely to resolve the discrepancy in outcome. Future studies should assess additional factors, including clinical status at presentation and barriers to accessing care, with the goal of identifying actionable mediating pathways. Mediation methods may be particularly helpful in this regard when the primary exposure is difficult or impossible to alter 10.

## Conflict of Interest

None declared.

## Supporting information


**Figure S1.** AAML0531 experimental design schema.
