# Supplementary figures and images for: Early discharge as a mediator of greater ICU‐level care requirements in patients not enrolled on the AAML0531 clinical trial: a Children's Oncology Group report
Source: Cancer Med. 2016 Jul 29;5(9):2412–6. doi: 10.1002/cam4.839 (PMC5055162; doi:10.1002/cam4.839)

**Supplementary Material**

**Supplemental Figure. AAML0531 Experimental Design Schema**

**
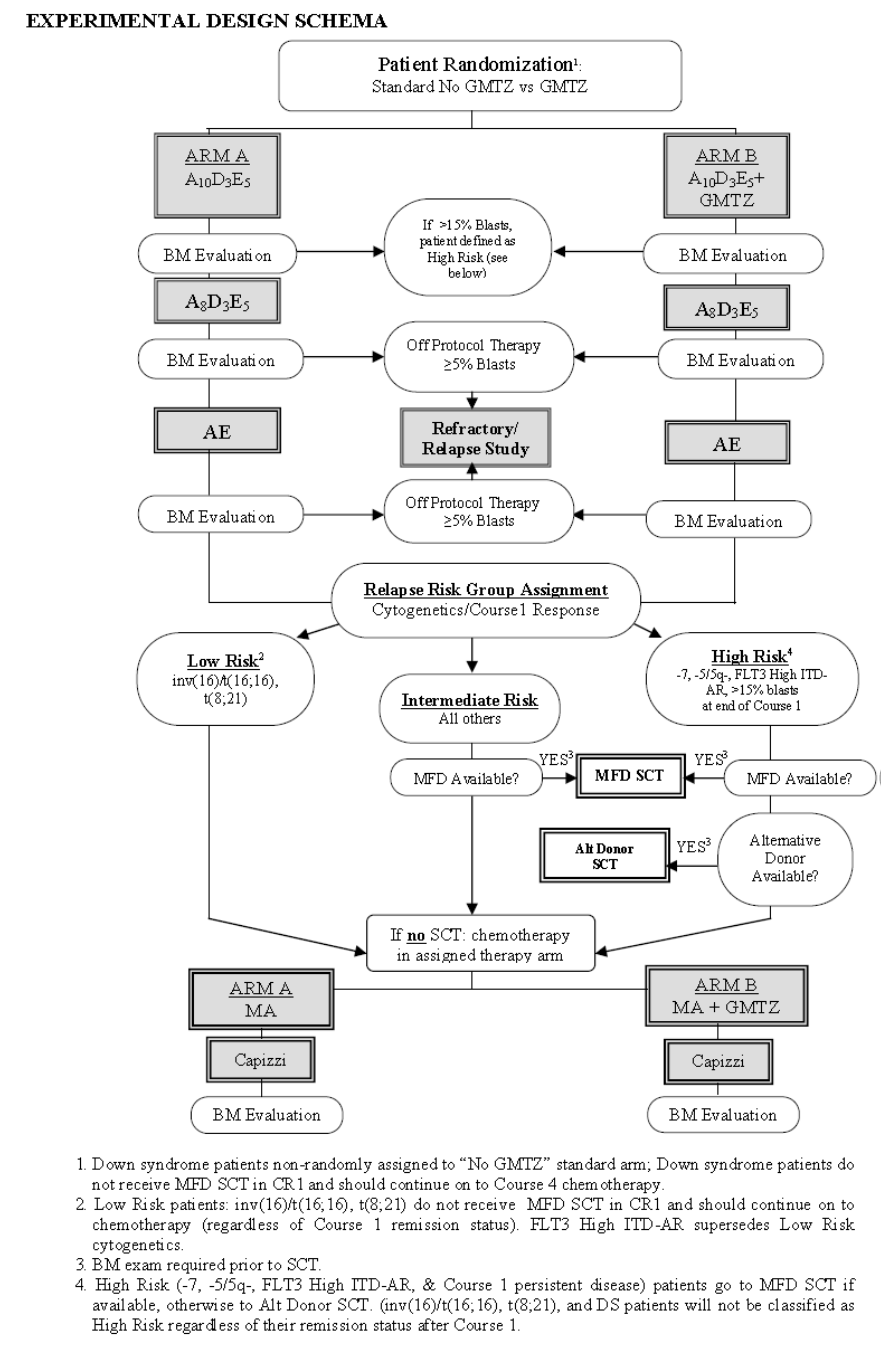
**

Supplement: Supplementary file 1 — Figure S1. AAML0531 experimental design schema. [file CAM4-5-2412-s001.docx]
